# HELIX Syndrome, a Claudinopathy with Relevant Dermatological Manifestations: Report of Two New Cases

**DOI:** 10.3390/genes15060687

**Published:** 2024-05-26

**Authors:** María Carmen Martínez-Romero, María Encarnación Hernández-Contreras, Juan Antonio Bafalliu-Vidal, María Barreda-Sánchez, Teresa Martínez-Menchón, Virginia Cabello-Chaves, Encarna Guillén-Navarro

**Affiliations:** 1 Molecular Genetics Section, Biochemistry and Clinical Genetics Center, University Clinical Hospital Virgen de la Arrixaca, 30120 Murcia, Spain; mcarmen.martinez16@carm.es (M.C.M.-R.); juana.bafalliu@carm.es (J.A.B.-V.); 2 Pediatric Research, Murcian Institute for Biosanitary Research (IMIB) Pascual Parrilla, 30120 Murcia, Spain; mbarreda@ucam.edu; 3 Centro de Investigación Biomédica en Red de Enfermedades Raras (CIBERER), Carlos III Health Institute, 28029 Madrid, Spain; 4 Faculty of Medicine, UCAM Catholic University of Murcia, 30109 Murcia, Spain; 5 Internal Medicine Service, University Clinical Hospital Virgen de la Arrixaca, 30120 Murcia, Spain; maencarna79@gmail.com; 6 Dermatology Department, University Clinical Hospital Virgen de la Arrixaca, 30120 Murcia, Spain; teresammenchon@gmail.com; 7 Nephrology Department, University Clinical Hospital Virgen del Rocío, 41013 Sevilla, Spain; vicacha@gmail.com; 8 Pediatrics Department, University Clinical Hospital Virgen de la Arrixaca, 30120 Murcia, Spain; 9 Surgery, Pediatrics, Obstetrics and Gynecology Department, University of Murcia (UMU), 30120 Murcia, Spain

**Keywords:** *CLDN10*, HELIX syndrome, hypohidrosis, ichthyosis, alacrimia, hypokalemia

## Abstract

HELIX syndrome (Hypohidrosis–Electrolyte disturbances–hypoLacrimia–Ichthyosis–Xerostomia) (MIM#617671) (ORPHA:528105), described in 2017, is due to an abnormal claudin 10 b protein, secondary to pathogenic *CLDN10* variants. So far, only ten families have been described. We aim to describe the phenotype in the first Spanish family identified, highlight the skin anomalies as an important clue, and expand the genotypic spectrum. Two adult brothers from consanguineous parents with suspected ectodermal dysplasia (ED) since early childhood were re-evaluated. A comprehensive phenotypic exam and an aCGH + SNP4 × 180 K microarray followed by Sanger sequencing of the *CLDN10* gene were performed. They presented hypohidrosis, xerosis, mild ichthyosis, plantar keratosis, palm hyperlinearity, alacrima, and xerostomia. In adulthood, they also developed a salt-losing nephropathy with hypokalemia and hypermagnesemia. The molecular study in both patients revealed a novel pathogenic homozygous deletion of 8 nucleotides in exon 2 of the *CLDN10* gene [*CLDN10* (NM_0006984.4): c.322_329delGGCTCCGA, p.Gly108fs*] leading to a premature truncation of the protein. Both parents were heterozygous carriers. Hypohidrosis, ichthyosis, and plantar keratosis associated with alacrima and xerostomia should raise suspicion for HELIX syndrome, which also includes nephropathy and electrolyte disturbances in adults. Given the potential for ED misdiagnosis in infancy, it is important to include the *CLDN10* gene in a specific genodermatosis next-generation sequencing (NGS) panel to provide early diagnosis, accurate management, and genetic counseling.

## 1. Introduction

The acronym HELIX (Hypohidrosis–Electrolyte disturbances–hypoLacrimia–Ichthyosis–Xerostomia) refers to a syndrome caused by changes in the claudin 10 b transcript (MIM#617671) (ORPHA:528105) [1]. Claudins are major components of tight junction (TJ) proteins expressed at the binding of adjacent epithelial and endothelial cells, establishing selective channels through membranes [2,3]. Defects in the *CLDN10* gene occur as an autosomal recessive disorder initially associated with salt-losing nephropathy with an imbalance in Ca^2+^ and Mg^2+^ homeostasis but also characterized by hypohidrosis, lacrimal gland dysfunction, ichthyosis, and xerostomia. The human *CLDN10* gene is located on chromosome 13q31-q34; its dysfunction was first described in 2017 and 35 cases from 10 different families worldwide have been reported in the literature [1,3,4,5,6,7,8]. Here, we report two cases of HELIX syndrome from the first Spanish family described, highlighting the dermatological manifestations as a clue for earlier diagnosis and adequate patient management.

## 2. Materials and Methods

Two brothers with suspected ectodermal dysplasia (ED) since early childhood, from Spanish consanguineous parents, were referred for clinical and molecular evaluation. Alacrimia was evaluated using a Schirmer test. Genetic analysis: first, an aCGH + SNP4 × 180 K microarray (Agilent Technologies, Wilmington, DE, USA) was performed to search for Copy Number Variants (CNVs) or loss of heterozygosity (LOH) in regions that could contain a gene suggestive of causing the phenotype observed in the patient. Next, Sanger sequencing of the *CLDN10* gene was performed as a second genetic test; primers for the *CLDN10* gene were designed, using design Primer3 plus open software, for all its coding exons and flanking sequences. Sequencing was performed with BigDye terminator chemistry (Life Technologies Carlsbad, CA, USA), followed by analysis in an ABI3500 Genetic Analyser (Applied Biosystems, Waltham, MA, USA) and alignment using the human reference genome GRCh37/hg19 version; the variant identified was segregated in the parents.

## 3. Results

### 3.1. Clinical Phenotype of the Cases Reported

**Figure 1:**
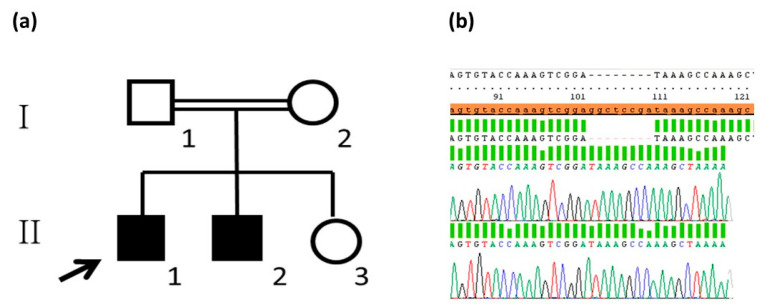
Family tree where generations are indicated in Roman numerals (I-II) and members of each generation are indicated in Arabic numerals (1–3) (**a**). Exon 2 sequence of the *CLDN10* gene in Case 1 with c.322_329 delGGCTCCGA; p.Gly108fs* in homozygosis (**b**).

The first patient was a 41-year-old male with a history of hypohidrosis and intolerance to exercise and high temperatures (Case 1). A brother of the patient (38 years old) had a similar clinical phenotype (Case 2). The parents of the patients were cousins; the mother, aunt, and maternal grandmother reported mild hypohidrosis without other associated symptoms, while the father and sister were asymptomatic (Figure 1a). Case 1 was diagnosed with hereditary simple congenital hypohidrosis at age 1, after presenting repeated febrile episodes, ruling out infections or metabolic diseases. Skin biopsy revealed dilated sweat glands with fewer secretion spots than in controls and significant papillary ridge hypoplasia. Rose Bengal and Schirmer tests showed abnormal results (2 mm in the right eye and less than 1 mm in the left eye); parotid and submandibular glandular gammagraphy were compatible with moderate to severe dysfunction. Due to an initial clinical suspicion of ED, a molecular study of the Ectodysplasin A (*EDA*) and Ectodysplasin A receptor (*EDAR*) genes had been requested during his childhood, as they are the most frequently implicated genes in this entity in the Spanish population [9]. The result was negative; thus, the involvement of other ED-related genes with autosomal recessive or X-linked inheritance patterns was suspected. Involvement of the *CLDN10* gene, associated with signs of ED and renal affectation, was suspected after observing LOH in the 13q31-q34 region using an aCGH SNP microarray. Subsequently, a new genetic molecular analysis was carried out by Sanger sequencing.

**Figure 2:**
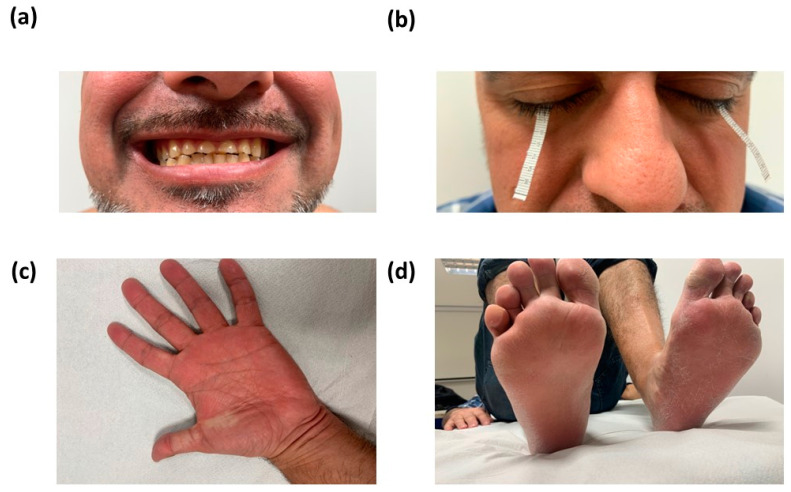
Phenotypic manifestations in Case 1: hypoplastic and severely impaired enamel wear of the permanent teeth with sawed edges (**a**), normal eye with alacrimia confirmed by a pathological Schirmer test (**b**), redness and hyperlinearity in the palms (**c**), xerosis, mild ichthyosis, and plantar keratosis (**d**).

In the reevaluation of Case 1, very dry skin with a tendency to flake, absence of oronasal secretions and tears, and frequent earplugs since childhood were reported as well as polyuria and polydipsia. For the last four years, he was followed up by nephrology due to elevated creatinine levels, related to chronic kidney disease, with normal blood pressure (122/77). During the examination, scalp, body hair, and nails were normal. Small teeth and enamel anomalies, xerosis, mild ichthyosis in legs, plantar keratosis, and palm hyperlinearity were noted (Figure 2).

Urine and blood biochemistry and hormone analysis showed salt-losing nephropathy with hypokalemia and hypermagnesemia, hypernatriuria, hypocalciuria, decreased glomerular filtration (ClCr < 60 mL/min), and elevation of creatinine and plasma aldosterone with normal plasma renin activity. His brother (Case 2) additionally presented multiple episodes of acute pericarditis, which had been managed with steroids and non-steroidal anti-inflammatory drugs (NSAIDs), and salt-losing tubulopathy with secondary hyperaldosteronism (Table 1).

### 3.2. Analysis of the Variant Detected

The analysis of the *CLDN10* gene by Sanger sequencing in Case 1 confirmed a pathogenic variant in homozygosis in exon 2 [*CLDN10* (NM_0006984.4): c.322_329delGGCTCCGA, p. Gly108fs*] (Figure 1b). This alteration involved a loss of 8 nucleotides that completely disrupted the second transmembrane helix of claudin-10 and prevented its correct function. This variant had not been observed in the control population (ExAC, genomAD, and 1000 genomes), and the *in silico* predictors indicated it to be pathogenic and associated with HELIX syndrome. The genetic study of the brother (Case 2) detected the same homozygous variant and the parents were heterozygous carriers; the asymptomatic sister refused to participate.

## 4. Discussion

To our knowledge, this is the first report of HELIX syndrome in Spanish patients referred with a suspicion of ED. It was caused by a novel homozygous pathogenic deletion in exon 2 of the *CLDN10* gene [(NM_0006984.4): c.322_329delGGCTCCGA, p. Gly108fs*]. The complete phenotype includes generalized hypohidrosis, with intolerance to high temperatures and physical exertion; xerosis, xerostomia, and ichthyosis; plantar keratoderma and palmar hyperlinearity with normal hair and nails; alacrima; severe alteration of tooth enamel with hypoplastic teeth; impaired renal function; polyuria and polydipsia and water and electrolyte imbalance, with mild hypokalemia, hypochloremia, and hypermagnesemia [3,4,5,6,7,8].

In comparison with other patients harboring previously described *CLDN10* alterations, two non-related European patients reported by Bongers et al., 2017 [4], had three different pathogenic variants in heterozygosis c.446C>G, p.Pro149Arg, c.465-1G>A, p.Glu157Tyr192del, and c.217G>A, p.Asp73Asn (Table 1). These two patients presented salt-losing nephropathy with hypokalemia, alkalosis, and hypochloremia (no Bartter or Gitelman) [10,11,12], with low urinary calcium concentrations and unexpectedly elevated serum levels of magnesium. These findings, similar to those found in our patients, are attributed to ion transport impairment in the thick ascending loop of Henle of the tubule [13,14]. However, no alterations at the skin and/or glandular level were reported and it is unknown whether they were overlooked or absent due to the double heterozygote character of the described variants.

The other patients reported in the literature were homozygous, with a related history of consanguinity [3,6] or not [1]. Hadj-Rabia et al., 2018 [1], were the first to coin the name HELIX, describing a pattern of xerostomia, hypohidrosis, muscle cramps with exercise, moderate xerosis, diffuse ichthyosis, alacrimia, keratoderma, palmar hyperlinearity, and tooth enamel alterations common to most cases. The patients described by Klar et al., 2017 [3], ranged between 27 and 47 years of age and also had generalized anhidrosis, severe heat intolerance, and renal failure. Four of these thirteen patients reported with the pathogenic variant c.144C>G, p. Asn48Lys in homozygosis had nephrolithiasis. The parathyroid hormone (PTH) value analyzed in two patients was high, suggesting the presence of secondary hyperaldosteronism. The case described by Meyers et al., 2019 [5], corresponded to a 12-year-old male from South America with a pathogenic homozygous variant, affected by polyuria, xerostomia, anhidrosis, and alacrimia, who also had hypokalemia, hypochloremia, hyperaldosteronism with normal plasma renin activity (similar to our case), and hypercholesterolemia. The case described by Alzahrani et al., 2021 [6], carried a frameshift homozygous variant, c.647delC, p. Pro 216Lys fs*21, causing premature protein truncation in both alleles, as in our patient. In addition, via functional studies, they showed mRNA degradation and protein retention in intracellular compartments [6] (Table 2).

In the most recent case reported by Sewerin et al., 2022 [8], an impaired enamel for-mation was noted as a new ectodermal feature in HELIX syndrome. Similar dental findings were observed in both brothers from the family described herein (see Case 1 in Figure 2a). Severe enamel wear could be attributed to decreased salivation; however, amelogenesis imperfecta has been lately related to a loss of *CLDN10* expression in the tooth. Immunostaining of developing tooth germs revealed that claudin-10 was highly expressed in murine inner enamel, suggesting that *CLDN10* could be a novel stratum intermedium marker and might play a role in the cytodifferentiation of the dental stratum intermedium [15].

To date, only eight pathogenic variants in *CLDN10* associated with HELIX syndrome or related disorders [10] have been reported in the professional human genomic mutation database of morbidity, the Human Gene Mutation Database (HGMD) [16]. We present an additional novel genotypic variant in the germline DNA of two Spanish brothers with a complete HELIX syndrome phenotype. They presented skin, gland, tooth, and kidney effects, which may be due to a more deleterious effect of this variant in homozygosity or a more comprehensive exam (Table 2).

Early diagnosis of these patients is critical to provide accurate genetic counseling and management, which should include skin and dental care and preventing exposure to extreme heat. Due to the predictable glomerular damage in the kidney, follow-up by nephrologists is mandatory. Current treatment is symptomatic and the prognosis in the long term is unknown; nevertheless, their quality of life can often be reduced by difficulties arising from environmental conditions or the body’s response to different infectious and/or metabolic processes.

Since dermatological manifestations present early in most HELIX syndrome patients and can be confused with potential ED conditions, mainly due to hypohidrosis, it would be important to include the *CLDN10* gene in specific genodermatosis multigene panels or to screen for it directly when hypermagnesemia and/or hypokalemia are also detected.

## Figures and Tables

**Table 1:** Laboratory results of Cases 1 and 2.

Laboratory Data	Case 1	Case 2	Reference Values
Age	41 years	38 years	
Serum values			
Plasma creatinine	1.60 mg/dL	1.14 mg/dL	0.70–1.20 mg/dL
Glomerular filtration (CKD-EPI mL/min)	52.73 mL/min/1.73 m^2^	82 mL/min/1.73 m^2^	>90 mL/min/1.73 m^2^
Plasma values			
Na^+^	141 mEq/L	normal	136–145 mEq/L
K^+^	3.3 mEq/L	2.5 mEq/L	3.5–5.1 mEq/L
Cl^−^	95 mEq/L	90 mEq/L	98–107 mEq/L
Mg^2+^	2.66 mEq/L	normal	1.60–2.60 mEq/L
Ca^2+^	9.4 mg/dL	9.5 mg/dL	8.6–10 mg/dL
Phosphorus	3.5 mg/dL	3.5 mg/dL	2.5–4.5 mg/dL
PTH	63.2 pg/mL	25 pg/mL	18.5–88 pg/mL
Aldosterone	348 pg/mL	NA	7.0–150 pg/mL
Renin	2.0 ng/mL/h	NA	0.8–2.1
Lipase activity	100 U/L	NA	13–69 U/L
Amylase	57 U/L	NA	13–53 U/L
25-OH vitamin D	11.3 μg/L	NA	30–100 μg/L
Blood pH	7.42	7.43	7.3–7.4
Bicarbonate	36.1 mEq/L	NA	22–26 mEq/L
Urine values			
Na^+^	161 mEq/L	NA	40–150 mEq/L
K^+^	29.1 mEq/L	NA	25–80 mEq/L
Cl^−^	138 mEq/L	>25 mEq	46–168 mEq/L
Mg^2+^	3.4 mg/dL	NA	25.7–131.9 mg/dL
Ca^2+^	0.9 mg/dL	<0.01 mg/dL	5–35 mg/dL

NA: not available. PTH: parathormone.

**Table 2:** Phenotype and genotype comparison in HELIX syndrome patients.

**References**	This Report	Meyers et al., 2019	Bongers et al., 2017	Klar et al., 2017	Hadj-Rabia et al., 2018	Alzahrani et al., 2021	Sewerin et al., 2022
Origin	Spain	Ecuador	The Netherlands	Pakistan	North Africa	Saudi Arabia	Afghanistan
Patients/Families	2/1	1/1	2/2	13/2	6/2	12/2	1/1
Genotype *CLDN10b* (NM_006984.4)	c.322_329del8 (p.G108fs*)	c.238A>G (p.R80G)	c.446C>G; c.465-1G>A (p.P149R; p.E157_Y192del) c.446C>G; c.217G>A (p.P149R; p.D73N)	c.144C>G (p.N48K)	c.2T>C (p.M1?) c.392C>T (p.S131L)	c.653delC (p.P218Lfs*21)	c.494C>G (p.G165A)
Hypohidrosis	2/2	1/1	ND	13/13	6/6	12/12	1/1
Lachrymal dysfunction	Yes	Yes	Yes	Yes	Yes	Yes	Yes
Xerosis, xerostomia	2/2	1/1	ND	13/13	6/6	12/12	0/1
Ichthyosis	2/2	0/1	ND	ND	6/6	11/12	0/1
Impairment of enamel wear	Yes	ND	ND	ND	Yes	ND	Yes
Hypokalemia	2/2	1/1	2/2	0/7	3/6	12/12	1/1
Hypermagnesemia	2/2	1/1	1/2	6/7	6/6	12/12	1/1
Abnormal glomerular filtration	2/2	1/1	1/2	0/3	1/6	12/12	1/1
Secondary hyperaldosteronism	Hyperaldosteronism w/o hyperreninism 2/2	Hyperaldosteronism w/o hyperreninism	ND	ND	6/6	12/12	Hyperaldosteronism w hyperreninism
Nephrolithiasis	0/2	0/1	0/2	4/13	0/6	ND	0/1
Intolerance to high temperatures and physical exercise	2/2	ND	ND	13/13	6/6	ND	ND
Disease reported	HELIX syndrome	HELIX syndrome	Salt-losing tubulopathy	Anhidrosis, heat intolerance, kidney damage	HELIX syndrome	HELIX syndrome	HELIX syndrome

ND: not described; w: with; w/o: without.

## Data Availability

The data presented in this study are available on request from the corresponding author. The data are not publicly available due to privacy restrictions.
